# Slow deformation event between large intraslab earthquakes at the Tonga Trench

**DOI:** 10.1038/s41598-020-80728-w

**Published:** 2021-01-08

**Authors:** Yuta Mitsui, Hinako Muramatsu, Yusaku Tanaka

**Affiliations:** 1 Department of Geosciences, Shizuoka University, Shizuoka, 422-8529 Japan; 2 Earthquake Research Institute, University of Tokyo, Tokyo, 113-0032 Japan

**Keywords:** Solid Earth sciences, Geodynamics, Geophysics, Seismology

## Abstract

Slow deformations associated with a subducting slab can affect quasi-static displacements and seismicity over a wide range of depths. Here, we analyse the seismotectonic activities in the Tonga subduction zone, which is the world’s most active area with regard to deep earthquakes. In our study, we combine data from global navigation satellite systems with an earthquake catalogue. We focus on the deep earthquakes that are below 400 km at the lower part of the Wadati–Benioff zone. We find that trenchward transient displacements and quiescence of deep earthquakes, in terms of background seismicity, were bounded in time by large intraslab earthquakes in 2009 and 2013. This “slow deformation event” between 2009 and 2013 may have been triggered by a distant and shallow M8.1 earthquake, which implies a slow slip event at the plate interface or a temporal acceleration of the subduction of the Pacific Plate. These findings provide new insights into the relationship between shallow and deep earthquakes in the subduction zone.

## Introduction

Several seismicity studies of subduction zones have recently suggested relationships between deep and shallow earthquakes as results of possible slow deformations^1,2^ or fluid ascent^3^. In particular, slow deformations associated with a subducting slab can affect quasi-static displacements and seismicity through changes in the stress state, like the variety of slow earthquakes that have been found at the Circum-Pacific subduction zones^4^, over a wide range of depths.

**Figure 1:**
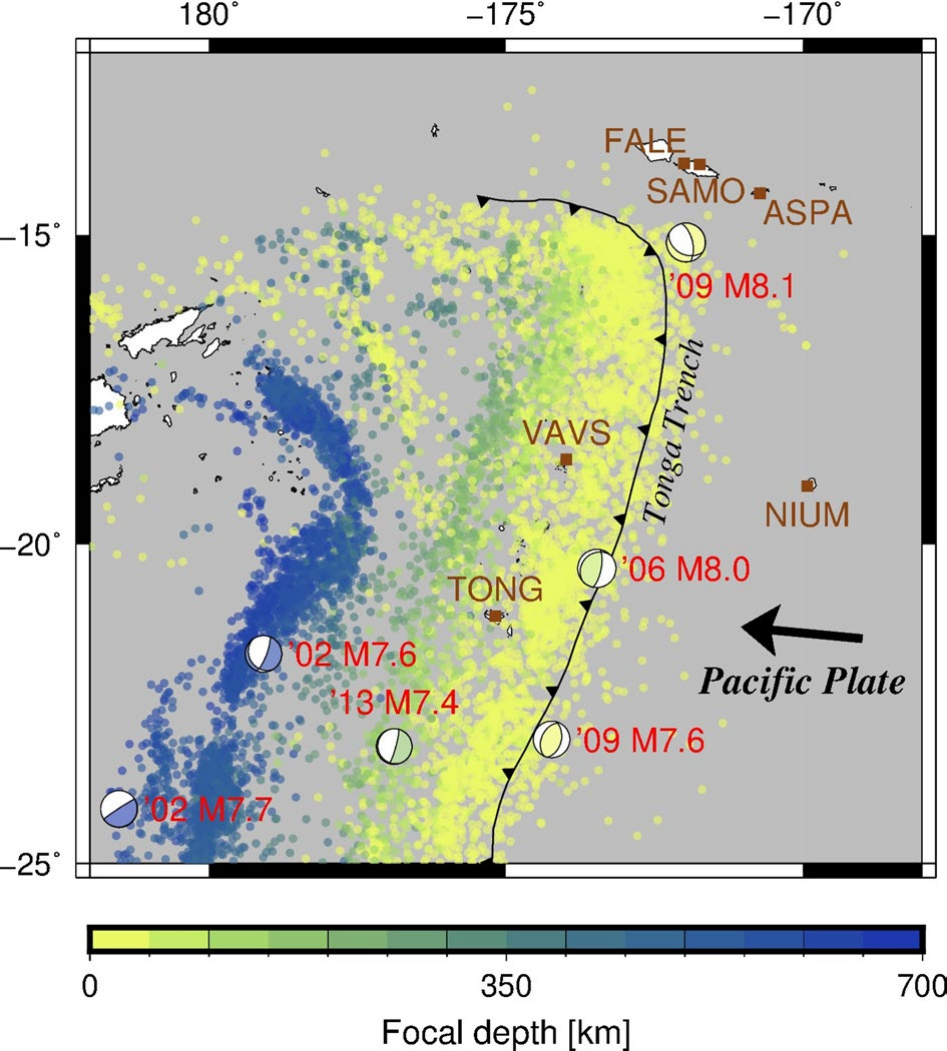
Seismicity map around the Tonga subduction zone, where the Pacific Plate subducts from the Tonga Trench. The coloured points show earthquakes during 2002–2017, the magnitudes of which are ≥ 4.5 as per the Advanced National Seismic System (ANSS) Comprehensive Earthquake Catalog (ComCat). Colour indicates focal depth. The beach-ball symbols and the red labels indicate the global Centroid Moment Tensor (CMT) solutions for large earthquakes, with magnitudes ≥ 7.4. The brown squares and labels indicate the Global Navigation Satellite System (GNSS) stations and their names. Figure generated with Generic Mapping Tools 5.x (https://www.generic-mapping-tools.org/).

The Tonga subduction zone, that is the most active area of deep earthquakes in the world^5^, belongs to the Circum-Pacific subduction zones. It is well known for frequent occurrences of earthquakes much larger than M7 (magnitude 7). Figure 1 is obtained from the global Centroid Moment Tensor (CMT) solutions^6^ and shows the seismicity map around the Tonga subduction zone. Among all the earthquakes, two deep intraslab earthquakes with dynamic triggering (M7.6 and M7.7) occurred on 19 August 2002^7^. Other shallow intraslab earthquakes occurred near the Tonga Trench on 3 May 2006 (M8.0) and 19 March 2009 (M7.6)^8^. A normal fault earthquake (M8.1) in the outer trench-slope occurred at the northern end of the Tonga subduction zone, possibly with two interplate thrust subevents, on 29 September 2009^9–11^. Figure 1 also shows that the seismicity at the lower part of the Wadati–Benioff zone, below 400 km (represented by blue colours), is extremely high. We will focus on these deeper earthquakes. This region is one of the best fields for investigating the relationship between deep earthquakes, shallow earthquakes, and slow deformations.

Monitoring the surface displacements using a Global Navigation Satellite System (GNSS) at the Tonga subduction zone revealed a rapid plate convergence (approximately 240 mm/year) and back-arc extension^12^. Although this region has few observation stations providing continuous GNSS monitoring, we can analyse temporal fluctuations of the surface displacements. Here, using GNSS time series and earthquake catalogue data, we analyse the seismotectonic activities in the Tonga subduction zone.

## Data

GNSS time series data in the ITRF2014/IGS14 Reference Frame^13^ provided by the Nevada Geodetic Laboratory^14^ allow us to analyse transient surface displacements at the Tonga subduction zones after 2002. The locations of the observation stations around the Tonga Trench are shown in Fig. 1.

We use Advanced National Seismic System (ANSS) Comprehensive Earthquake Catalog (ComCat) data^15^ for the timespan of 2002–2017 and the spatial range of 12–25 degrees south latitude and 178–192 degrees east longitude (Fig. 1). We exclude earthquakes of M < 4.5 based on a histogram of earthquake magnitudes (Supplementary Fig. S1). The magnitude value of 4.5 is a standard threshold for earthquake statistics studies using the ANSS ComCat.

**Figure 2:**
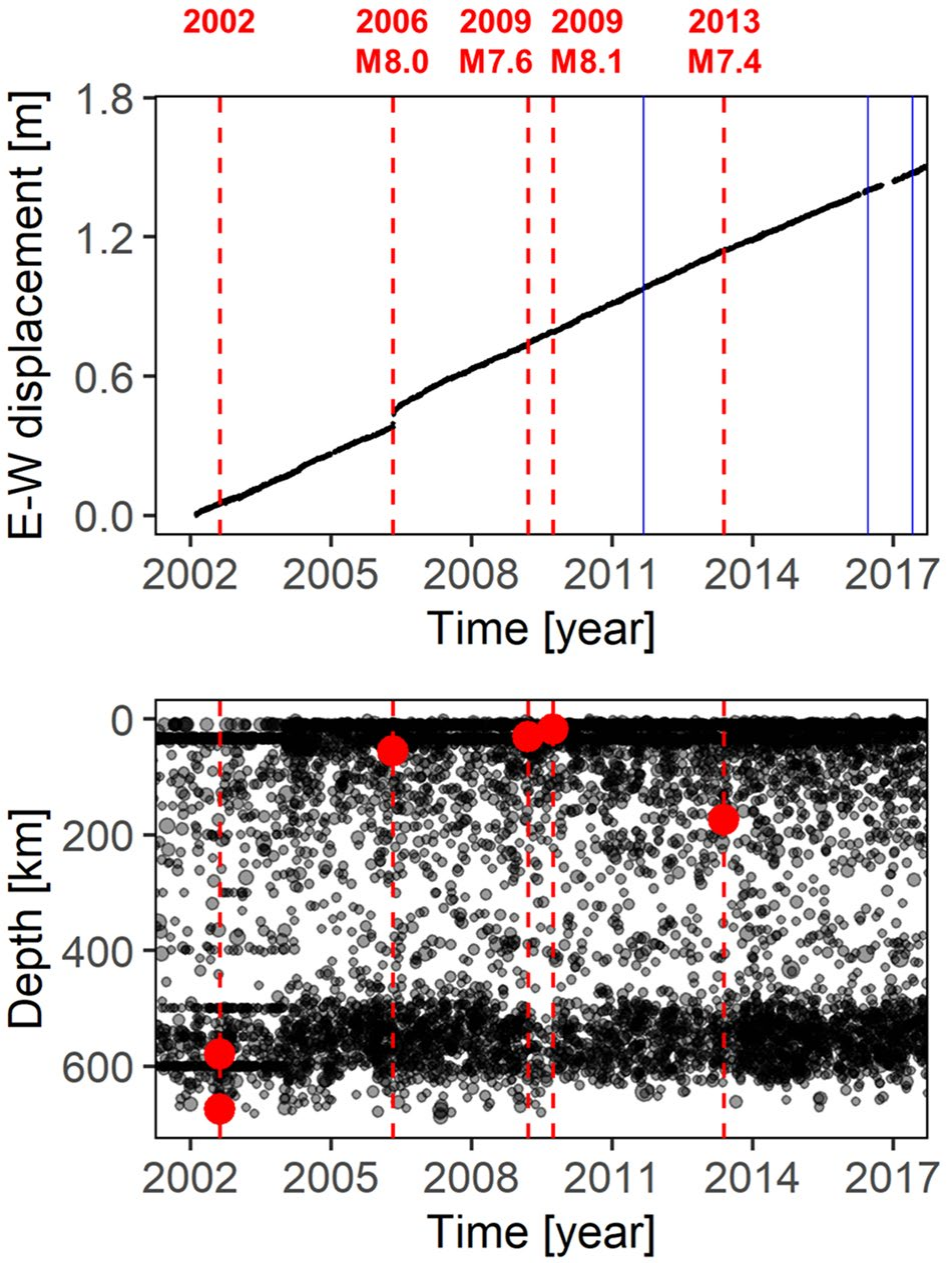
(Top) Example of the GNSS time series data (East–West component at TONG station). (Bottom) Depth-time evolution of the seismicity. The red vertical broken lines and the red circles indicate the occurrences and the focal depths of the respective large earthquakes in Fig. 1. The vertical blue lines indicate the time of the equipment changes for the GNSS station. Figures generated with R 3.x (https://www.r-project.org/).

Figure 2 shows the results for an example of the GNSS time series (East–West component at TONG station) and the depth time evolution of the earthquakes. The time series reveals stable data acquisition. The depth time evolution of the earthquakes suggests that older data, those before ca. 2004, fixed the focal depths (e.g., 450 km) of several deep earthquakes.

## Methods

### GNSS data analysis

To extract signals of transient surface displacements in the GNSS time series, we first remove the offsets reflecting equipment changes for the GNSS stations by estimating the differences in median values on five days before and after the offset times. If the terms for the offset estimation are separated by more than two weeks, we do not perform the offset correction.

Then, we detrend the time series focusing on the transient displacements after the M8-class large shallow earthquakes (on 3 May 2006 and 29 September 2009). Namely, the detrended time series data are obtained by removing linear trends in the one-year period preceding the 2006 earthquake. Supplementary Fig. S2 shows the detrended GNSS time series at all the stations.

### Earthquake catalogue analysis

To exclude the effects of aftershock clustering from the earthquake catalogue data, we use the Epidemic-Type Aftershock Sequence (ETAS) model to extract the background seismicity rate $\mu$^16^. The ETAS model separates the seismicity rate into the background seismicity rate $\mu$ and clusters of aftershocks. The seismicity rate $\lambda \left(t\right)$ at time $t$ is given by

$$\lambda \left(t\right)=\mu +\sum_{{t}_{i}\le t}\frac{K {e}^{\alpha \left({M}_{i}-{M}_{c}\right)}}{(t-{t}_{i}+c{)}^{p}}(1)$$

where ${M}_{i}$ and ${t}_{i}$ are the magnitude and time, respectively, of the $i$-th earthquake, ${M}_{c}$ is the minimum magnitude (= 4.5), and the other parameters ($K$, ${\upalpha }$, $c$, and $p$) are constants. The five parameters in logarithmic form (log $\mu$, log $K$, log ${\upalpha }$, log $c$, and log $p$) are estimated by a limited-memory modification of the quasi-Newton method with box constraints^17^.

We perform the ETAS analysis on yearly data. For the parameter estimation, the initial values are assumed to be ($\mu$, $K$, ${\upalpha }$, $c$, $p$) = (0.1, 1, 1, 1, 1) and their optimal values are assumed to be in the ranges of 0.001–10, 0.01–100, 0.01–100, 0.01–100, and 0.01–100, respectively.

## Results

### Surface displacements

On the basis of the detrended GNSS time series (Supplementary Fig. S2), we confirmed that the two GNSS stations on the upper plate (TONG and VAVS) detected the large deformations related to the 2006 M8.0 earthquake, and the four stations on the lower Pacific Plate (NIUM, FALE, SAMO, and ASPA) detected the large deformations related to the 2009 M8.1 earthquake, as shown by a previous study^10^.

**Figure 3:**
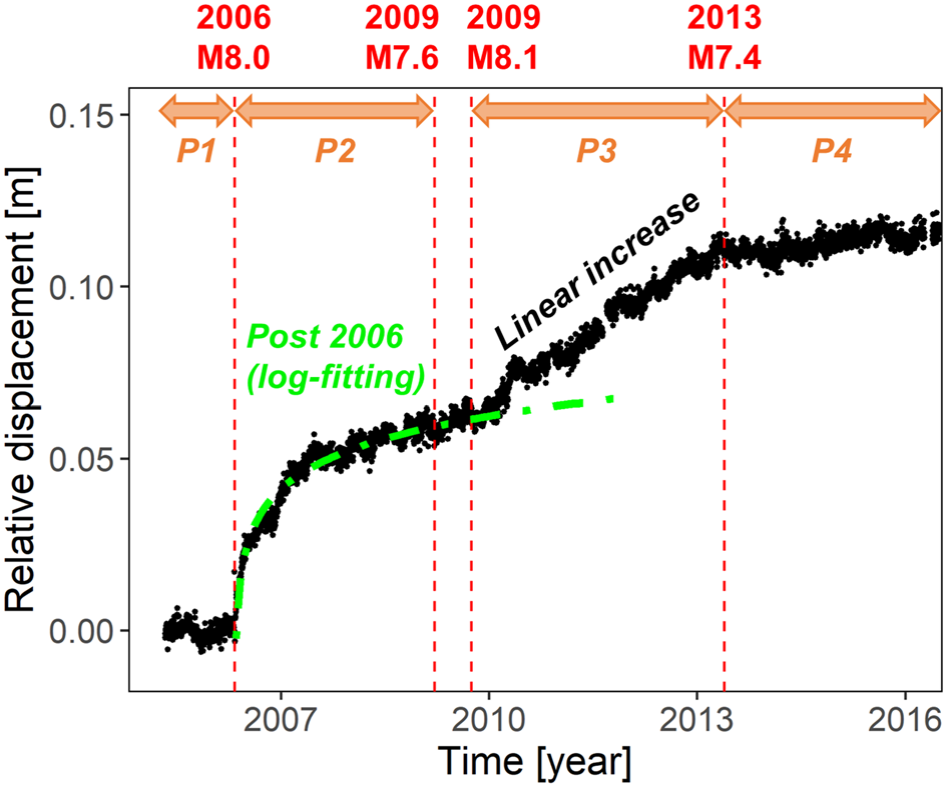
Detrended GNSS time series of the East–West component at TONG station, where we removed the coseismic offsets of the large earthquakes (vertical red broken lines) in Fig. 1. We define four periods, P1, P2, P3, and P4, bounded by the time of the large earthquakes. During P2, the logarithmic decay of the eastward displacements fitted by the green chain line (the relaxation time is about 0.024 years, equal to 8.8 days) was observed. During P3, the eastward displacements linearly increased. Figure generated with R 3.x (https://www.r-project.org/).

Here, we focus on the behaviour of the southernmost TONG station on the upper plate, of which transient displacements were not investigated in the previous study^10^. Figure 3 shows the detrended time series at the TONG station, where we removed the coseismic offsets of the large earthquakes using a similar method to that for the equipment changes. We define four periods: P1, P2, P3, and P4. P1 (before the 2006 earthquake) is the reference period for the detrending process. This figure shows that the displacement rate during P4 (after the 2013 earthquake) is similar to that during P1. This suggests that the transient surface displacements following the M8-class shallow earthquakes (on 3 May 2006 and 29 September 2009) occurred mostly during P2 and P3.

For P2 in Fig. 3, the transient displacements can be well fitted by a logarithmic function as $d\left(t\right)=0.012\mathrm{ln}(t/0.024),$ where $d\left(t\right)$ means the displacements in the past year $t$ from the beginning of P2 (the 2006 M8.0 earthquake). Such logarithmic behaviour is typical for postseismic deformation of large earthquakes^18^ and may reflect postseismic relaxation following bi-viscous Burgers rheology^19^. Besides, we confirmed that the relative amount of the transient displacements during P2 (~ 5.9 cm) to that of the coseismic offset of the 2006 M8.0 earthquake (~ 5.8 cm) as shown in Supplementary Fig. S2, was about 1. This value is not surprising, given the transient displacements during P2 was normal postseismic deformation of the 2006 M8.0 earthquake, compared with previous studies for postseismic deformation^20–22^.

In contrast, for P3, the transient displacements linearly increased from the beginning of P3 (the 2009 M8.1 earthquake) and suddenly ceased around the 2013 M7.4 earthquake (Fig. 3), which is the boundary between P3 and P4. The amount of transient displacements during P3 (~ 4.8 cm) was extremely larger than that of the coseismic offset of the 2009 M8.1 earthquake (~ 0 cm) as shown in Supplementary Fig. S2 These findings reveal that the transient displacements during P3 were not simple postseismic deformation of the 2009 M8.1 earthquake. For the other station on the upper plate, VAVS, we also find that the transient displacements during P3 differed from the rapid logarithmic decay of the postseismic deformation during P2 (Supplementary Fig. S3). The transient displacements on the upper plate during P3 were not ordinary postseismic deformation.

**Figure 4:**
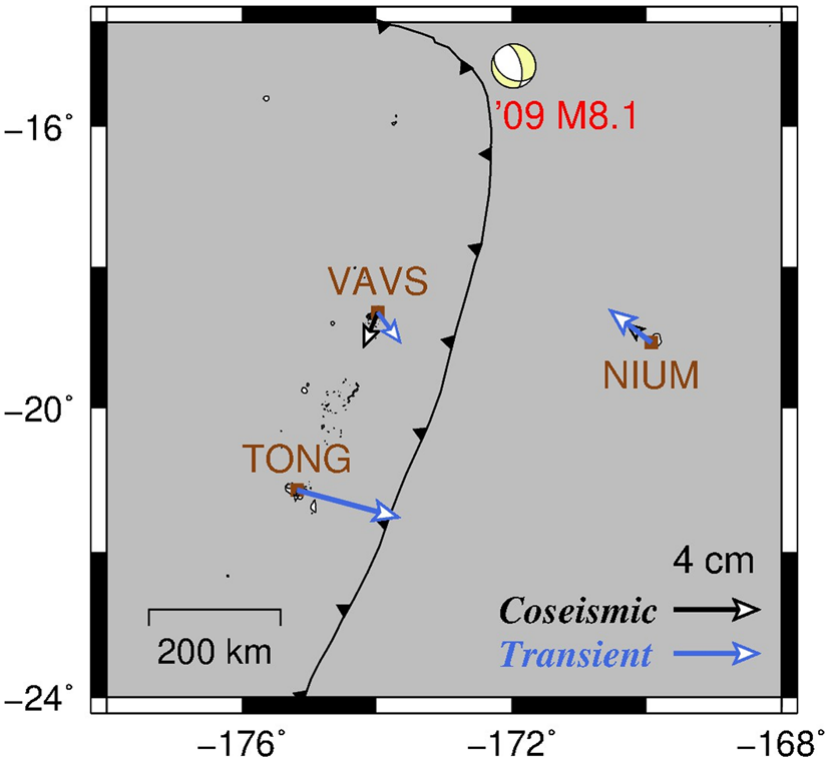
Displacement vectors associated with the M8.1 earthquake on 29 September 2009. The coseismic (black arrows) and transient displacements during period P3 (blue arrows) are mapped. Figure generated with Generic Mapping Tools 5.x (https://www.generic-mapping-tools.org/).

Furthermore, Fig. 4 shows the displacement vectors associated with the 2009 M8.1 earthquake around the upper plate stations (and the southern part of the Tonga subduction zone). The transient displacements during P3 at the TONG station were almost trenchward, and were larger than the displacements at the other stations (VAVS and NIUM) even though the TONG station was the most distant observation point (~ 750 km) from the source of the 2009 M8.1 earthquake.

### Earthquake activity

**Figure 5:**
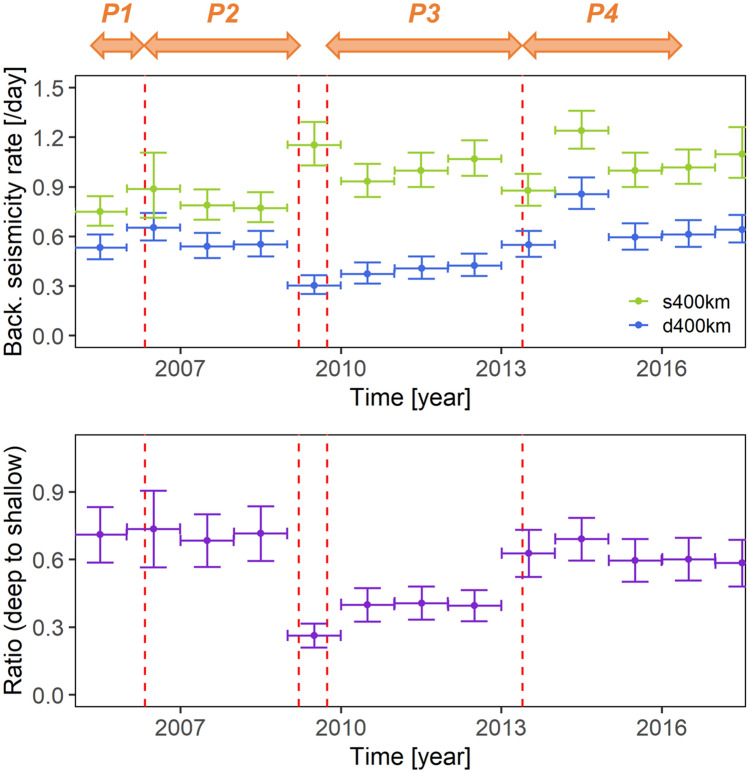
(Top) Temporal evolution of the background seismicity rate $\mu$ for earthquakes, with focal depths shallower than 400 km (s400km) and deeper than 400 km (d400km), as obtained from Epidemic-Type Aftershock Sequence (ETAS) analysis. The vertical error bars represent 95% confidence intervals. As in the lower panel of Fig. 2, the red vertical broken lines indicate the time of the large earthquakes. The four periods, P1, P2, P3, and P4, are shown by the orange arrows for reference. (Bottom) Temporal evolution of the ratio of the background seismicity rate $\mu$ for the deeper earthquakes (d400km) to that for the shallower earthquakes (s400km). Figures generated with R 3.x (https://www.r-project.org/).

Figure 5 shows the result of the ETAS analysis. The background seismicity rate $\mu$ of the deeper earthquakes, the focal depths of which were deeper than 400 km (d400km), decreased by a few tens of a percent from the end of P2 (probably for the 2009 M7.6 earthquake, not the 2009 M8.1 earthquake) to the end of P3. By contrast, the background seismicity rate $\mu$ of shallower earthquakes with a focal depth less than 400 km (s400km) showed slight increases from the end of P2.

In order to extract the characteristics of the deeper and shallower earthquake activities, we took the ratio of the background seismicity rate $\mu$ of the deeper earthquakes (d400km) to that of the shallower earthquakes (s400km). This ratio (Fig. 5) revealed that the quiescence period of the deeper earthquakes finished at approximately the same time around the 2013 M7.4 earthquake. The transient displacements (Fig. 3) ceased as well.

## Discussion

The trenchward transient displacements at the upper plate, which are measured by the TONG and VAVS stations (Fig. 4), were not investigated in the previous study^10^. The mentioned study modelled the viscoelastic relaxation of the 2009 M8.1 earthquake to explain the postseismic deformation. It was based on the GNSS and Gravity Recovery And Climate Experiment (GRACE) data. In another region of the central Kuril Islands with near-trench major earthquakes^23^, no additional trenchward movement similar to that estimated in this study was reported^24^.

Here, in order to compare the sophisticated model results of viscoelastic relaxation of the 2009 M8.1 earthquake with our results, we perform the same simulation as the previous study^10^ using the same numerical code VISCO1D^25^. Here is a brief overview of that simulation: The code calculates viscoelastic relaxation of the asthenosphere following the earthquake on a layered spherical Earth. The 2009 M8.1 earthquake was modelled as a combination of normal and thrust faultings (Supplementary Table S1). The thickness of elastic lithosphere is 62 km, and the viscoelastic asthenosphere extends from a depth of 62–220 km. We assume a bi-viscous Burgers body for the asthenosphere with the Maxwellian viscosity of $2\times {10}^{18}$ Pa s and the Kelvin viscosity of $1\times {10}^{17}$ Pa s. Moreover, the Maxwellian viscosity for the upper (220–670 km) and lower (670–2900 km) mantle are set to be $1\times {10}^{20}$ Pa s and $1\times {10}^{21}$ Pa s. The other parameters are described in Supplementary Table S2.

**Figure 6:**
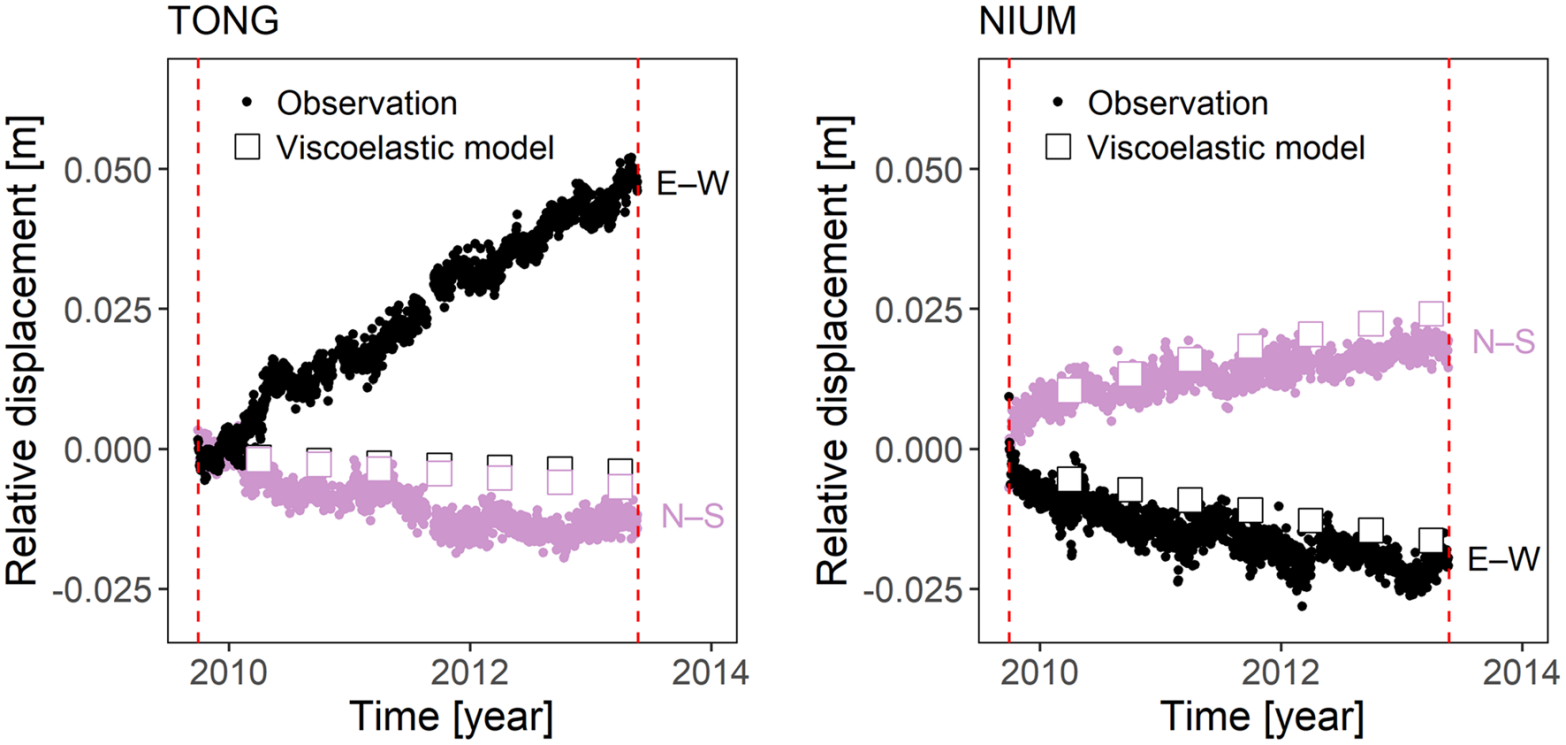
Comparison between the GNSS observation and the simulation results of the viscoelastic model in the previous study^10^ during period P3, at the TONG station (left) and the NIUM station (right). Figures generated with R 3.x (https://www.r-project.org/).

Figure 6 shows a comparison between the GNSS observations and the viscoelastic simulation during P3 at the southern part of the Tonga subduction zone. This comparison confirms that the transient displacements at the TONG station (previously shown in Figs. 3, 4) cannot be explained by the viscoelastic relaxation. This finding supports our prior conclusion that the transient displacements at the TONG station during P3 (Figs. 3, 4) were not ordinary postseismic deformation of the 2009 M8.1 earthquake. By contrast, Fig. 6 also shows that the displacements at the lower plate NIUM station after the 2009 M8.1 earthquake well followed the viscoelastic simulation as investigated in the previous study^10^.

**Figure 7:**
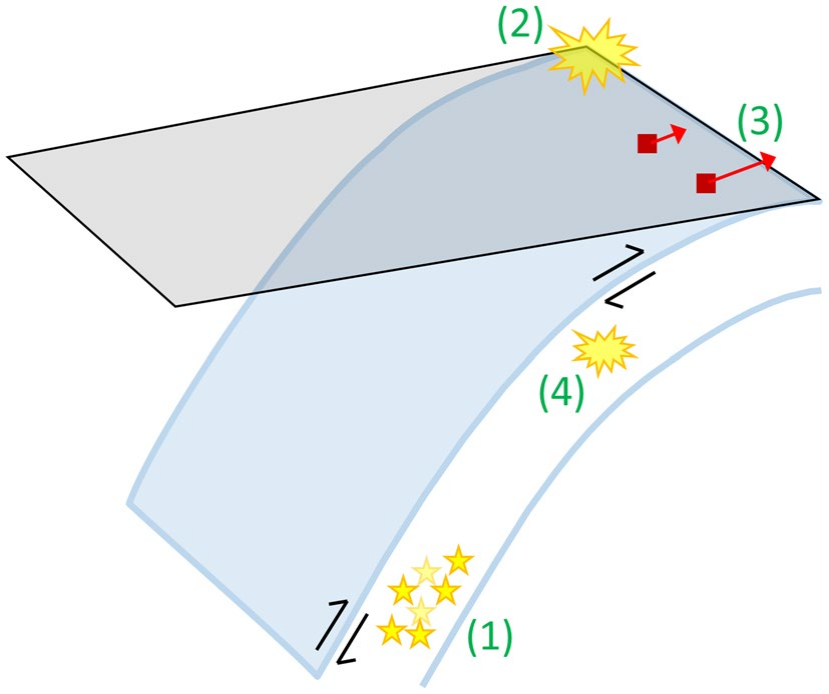
Illustration of a series of events at the southern part of the Tonga subduction zone after 2009. (1) Beginning of the quiescence of the deeper earthquakes. (2) The 2009 M8.1 shallow earthquake in the far north. (3) Beginning of the trenchward displacements at the upper plate TONG and VAVS stations (red arrows). (4) The 2013 M7.4 intraslab earthquake, and the end of the above “slow deformation event”.

To sum up, what happened in the Tonga subduction zone? During P2, after the 2006 M8.0 earthquake, we observed the typical logarithmic decay of postseismic deformation at the GNSS stations (e.g., Fig. 3) and the nearly constant (equal to those in the previous P1) background seismicity rates (Fig. 5). On the contrary, the quiescence of the deeper earthquakes started around 2009 (Fig. 5), and the trenchward transient displacements at the upper plate GNSS stations in the southern part of the Tonga subduction zone (e.g., the linear increase in Fig. 3) occurred after the 2009 M8.1 shallow earthquake in the far north as P3. During P3, before the 2013 M7.4 earthquake, the background seismicity rates slowly returned to the same state as before 2009. The trenchward transient displacements also ceased around the 2013 M7.4 earthquake. Figure 7 illustrates the above event series after 2009. Further, we refer to both the deep earthquake quiescence and the trenchward transient displacements together as a “slow deformation event”.

During the slow deformation event, the trenchward displacements, whuch cannot be explained by the viscoelastic relaxation mechanism, were triggered by stress changes due to the 2009 M8.1 intraslab earthquake in the far north through the following possible processes: (i) A slow slip event at the plate interface was triggered as in the case of the 2016 M7.8 Kaikoura earthquake in New Zealand^26^ or (ii) temporal acceleration of the subduction of the Pacific Plate to recover the force balance of plate tectonics was triggered and induced the changes in the displacement field, as in the case of the 2003 M8.0 Tokachi-oki earthquake in Japan^27^. Unfortunately, it is difficult to distinguish between the two possible processes from the present data set. We expect that the GRACE and its follow-on data will contribute to solving this problem.

The mechanisms for the slow deformation event that finished around the time of the 2013 M7.4 intraslab earthquake are not yet understood. One speculation is that the slab deformations due to the 2013 M7.4 earthquake regained the force balance before the event series. Besides, the possible effects of sudden fluid exchange^28^ or densification of metastable slab^29^ might cause the large-scale phenomena. In any case, the findings of the present study provide new insights into the relationship between shallow and deep earthquakes in the subduction zone.

## Conclusion

At the Tonga subduction zone, we identified a slow deformation event of the trenchward transient displacements and the quiescence of the deeper earthquakes. The time-related accordance of both phenomena and the large intraslab earthquakes implied an interconnected system between earthquakes from shallow to deep parts of the subduction zone.

## Supplementary Information

Supplementary Information.
