# Supplementary material for: Slow deformation event between large intraslab earthquakes at the Tonga Trench
Source: Sci Rep. 2021 Jan 8;11:257. doi: 10.1038/s41598-020-80728-w (PMC7794577; doi:10.1038/s41598-020-80728-w)
Supplement: Supplementary file 1 — Supplementary Information. [file 41598_2020_80728_MOESM1_ESM.docx]

Supplementary Information

**Slow deformation event between large intraslab earthquakes
at the Tonga Trench**

Yuta Mitsui, Hinako Muramatsu, Yusaku Tanaka

**Contents of this file**

Figure S1 shows changes of cumulative histograms of earthquake magnitudes ($\geq4.5$) every five years for the spatial range of 12-25 degrees south latitude and 178-192 degrees east longitude, as shown by Figure 1. All of the histograms have almost linear decreasing trends, which mean close to constant conditions for the detection of earthquakes following the Gutenberg-Richter law (Gutenberg and Richter, 1944).

Figure S2 illustrates detrended GNSS time-series at all the stations. We did not remove the coseismic offsets of the large earthquakes in Figure 1, in order to show the amounts of the coseismic and postseismic displacements.

Figure S3 presents detrended GNSS time-series of East-West component at VAVS station, with removing the coseismic offsets of the large earthquakes like Figure 3 in the manuscript. Unfortunately there is critical lack of the data around the 2009 M7.6 earthquake due to equipment change.

　Tables S1 and S2 describe source and structural parameters (other than viscosities which are written in the main text) for the viscoelastic relaxation model of the 2009 M8.1 earthquake (Han et al., 2019). The elastic parameters in Table S2 are consistent with Preliminary Reference Earth Model (Dziewonski & Anderson, 1981).

**Reference**

Dziewonski AM, Anderson DL. (1981) Preliminary reference Earth model. Phys Earth Planet Int, 25(4), 297–356.

Gutenberg B, Richter CF. (1944) Frequency of earthquakes in California. Bull Seis Soc Am, 34, 185–188.

Han S, Sauber J, Pollitz F, Ray R. (2019) Sea level rise in the Samoan Islands escalated by viscoelastic relaxation after the 2009 Samoa-Tonga earthquake. J Geophys Res, 124, 4142–4156.


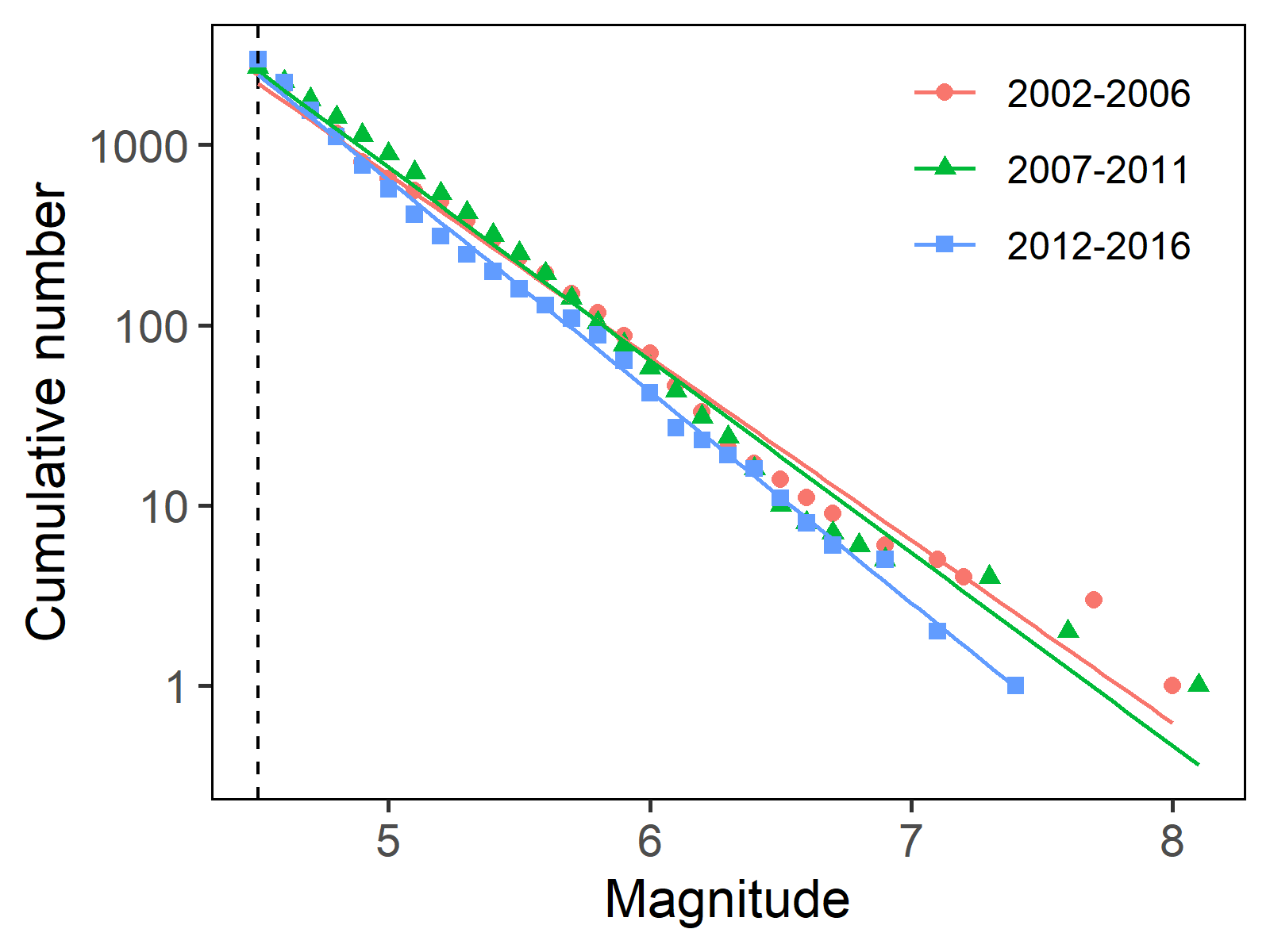


**Figure S1.**  Cumulative histograms of earthquake magnitudes (≥4.5) every five years for the same spatial range of Figure 1. The vertical broken line indicates M4.5. Figure generated with R 3.x (https://www.r-project.org/).


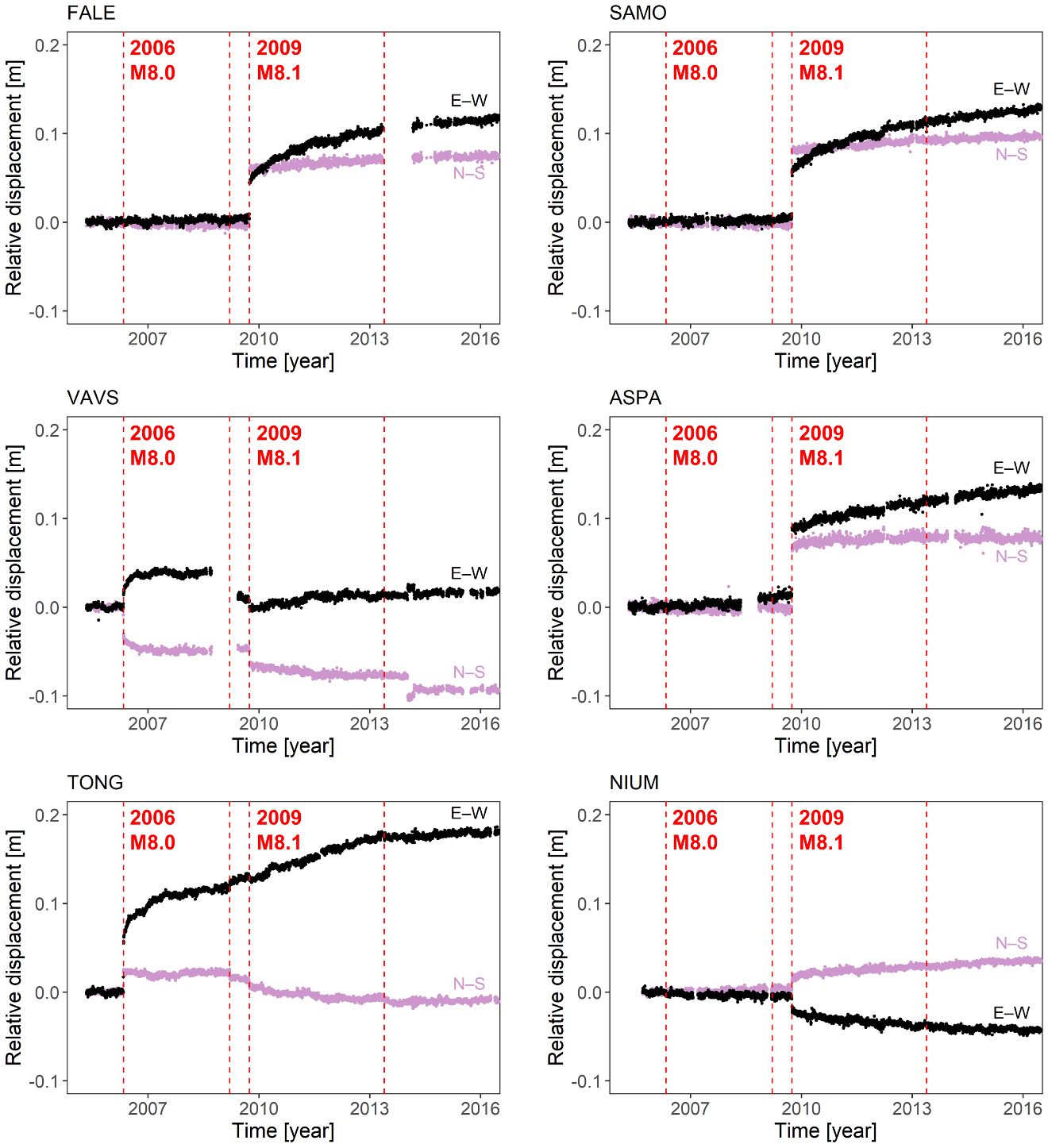


**Figure S2.**  Detrended time-series data at all the GNSS stations, where we did not remove the coseismic offsets of the large earthquakes (vertical red broken lines) in Figure 1. The black and pink points represent the E-W (East-West) and N-S (North-South) components, respectively. Figures generated with R 3.x (https://www.r-project.org/).


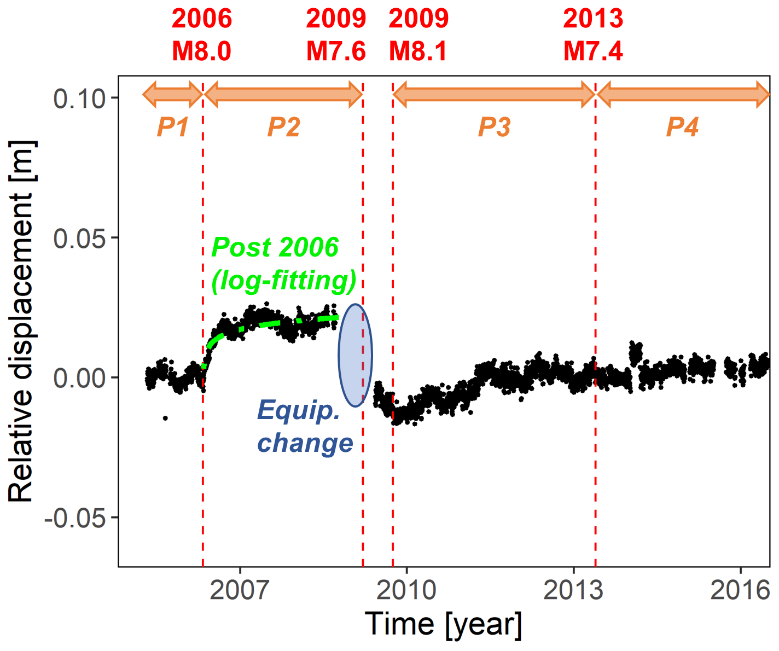


**Figure S3.**  Detrended time-series data of East-West component at VAVS station, where we removed the coseismic offsets of the large earthquakes (vertical red broken lines), like Figure 3 in the manuscript. Note that there is critical lack of the data around the 2009 M7.6 earthquake due to equipment change and we did not remove its offset. Figures generated with R 3.x (https://www.r-project.org/).

**Table S1.**  Source (fault) parameters for the viscoelastic relaxation model (Han et al., 2019). The latitude and longitude represent the coordinate at the lowermost corner of the fault closest to the strike direction.

| Lower fault edge depth  [km] | Upper fault edge depth  [km] | Dip  [deg.] | Latitude  [deg.] | Longitude  [deg.] | Length  [km] | Strike  [deg.] | Rake  [deg.] | Slip  [cm] |
| --- | --- | --- | --- | --- | --- | --- | --- | --- |
| 59.9 | 14.9 | 30 | -16.4310 | -173.0077 | 109 | 175 | 80 | 477 |
| 20.8 | 0.0 | 48 | -15.0285 | -172.1526 | 114 | 352 | -43 | 1067 |

**Table S2.**  Structural parameters for the viscoelastic relaxation model (Han et al., 2019).

| Layer | Bottom radius  [km] | Top radius  [km] | Density  [g/cm^3^] | Bulk modulus  [10^10^ Pa] | Maxwellian  shear modulus  [10^10^ Pa] | Kelvin  shear modulus  [10^10^ Pa] |
| --- | --- | --- | --- | --- | --- | --- |
| 1 | 3480 | 3481 | 5.566 | 44 | 21.91 | － |
| 2 | 3481 | 3500 | 5.536 | 44 | 21.91 | － |
| 3 | 3500 | 3600 | 5.506 | 44 | 21.91 | － |
| 4 | 3600 | 3700 | 5.497 | 44 | 21.91 | － |
| 5 | 3700 | 3800 | 5.491 | 44 | 21.91 | － |
| 6 | 3800 | 3900 | 5.357 | 44 | 21.91 | － |
| 7 | 3900 | 4000 | 5.307 | 44 | 21.91 | － |
| 8 | 4000 | 4100 | 5.257 | 44 | 21.91 | － |
| 9 | 4100 | 4200 | 5.207 | 44 | 21.91 | － |
| 10 | 4200 | 4300 | 5.156 | 44 | 21.91 | － |
| 11 | 4300 | 4400 | 5.105 | 44 | 21.91 | － |
| 12 | 4400 | 4500 | 5.054 | 44 | 21.91 | － |
| 13 | 4500 | 4600 | 5.003 | 44 | 21.91 | － |
| 14 | 4600 | 4700 | 4.95 | 44 | 21.91 | － |
| 15 | 4700 | 4800 | 4.897 | 44 | 21.91 | － |
| 16 | 4800 | 4900 | 4.843 | 44 | 21.91 | － |
| 17 | 4900 | 5000 | 4.789 | 44 | 21.91 | － |
| 18 | 5000 | 5100 | 4.733 | 44 | 21.91 | － |
| 19 | 5100 | 5200 | 4.678 | 44 | 21.91 | － |
| 20 | 5200 | 5319.1 | 4.67 | 44 | 21.91 | － |
| 21 | 5319.1 | 5353.7 | 4.635 | 44 | 21.91 | － |
| 22 | 5353.7 | 5388.3 | 4.611 | 44 | 21.91 | － |
| 23 | 5388.3 | 5422.9 | 4.587 | 44 | 21.91 | － |
| 24 | 5422.9 | 5457.6 | 4.563 | 44 | 21.91 | – |
| 25 | 5457.6 | 5492.2 | 4.539 | 44 | 21.91 | － |
| 26 | 5492.2 | 5526.8 | 4.515 | 44 | 21.91 | － |
| 27 | 5526.8 | 5561.5 | 4.491 | 44 | 21.91 | － |
| 28 | 5561.5 | 5596.1 | 4.467 | 44 | 21.91 | － |
| 29 | 5596.1 | 5630.7 | 4.443 | 44 | 21.91 | － |
| 30 | 5630.7 | 5665.4 | 4.443 | 44 | 21.91 | － |
| 31 | 5665.4 | 5701 | 4.38 | 44 | 21.91 | － |
| 32 | 5701 | 5731.3 | 3.992 | 19 | 9.8 | － |
| 33 | 5731.3 | 5771 | 3.983 | 19 | 9.8 | － |
| 34 | 5771 | 5793.8 | 3.975 | 19 | 9.8 | － |
| 35 | 5793.8 | 5825 | 3.931 | 19 | 9.8 | － |
| 36 | 5825 | 5856.3 | 3.89 | 19 | 9.8 | － |
| 37 | 5856.3 | 5887.5 | 3.849 | 19 | 9.8 | － |
| 38 | 5887.5 | 5918.7 | 3.764 | 19 | 9.8 | － |
| 39 | 5918.7 | 5950 | 3.743 | 19 | 9.8 | － |
| 40 | 5950 | 5971 | 3.723 | 19 | 9.8 | － |
| 41 | 5971 | 6001.2 | 3.543 | 19 | 9.8 | － |
| 42 | 6001.2 | 6026.9 | 3.543 | 19 | 9.8 | － |
| 43 | 6026.9 | 6052.5 | 3.49 | 19 | 9.8 | － |
| 44 | 6052.5 | 6078.1 | 3.49 | 19 | 9.8 | － |
| 45 | 6078.1 | 6103.8 | 3.435 | 19 | 9.8 | － |
| 46 | 6103.8 | 6129.4 | 3.435 | 19 | 9.8 | － |
| 47 | 6129.4 | 6151 | 3.435 | 19 | 9.8 | － |
| 48 | 6151 | 6180.6 | 3.36 | 13 | 6.7 | 6.7 |
| 49 | 6180.6 | 6206.3 | 3.36 | 13 | 6.7 | 6.7 |
| 50 | 6206.3 | 6231.9 | 3.367 | 13 | 6.7 | 6.7 |
| 51 | 6231.9 | 6257.5 | 3.367 | 13 | 6.7 | 6.7 |
| 52 | 6257.5 | 6283.1 | 3.37 | 13 | 6.7 | 6.7 |
| 53 | 6283.1 | 6308.8 | 3.374 | 13 | 6.7 | 6.7 |
| 54 | 6308.8 | 6321 | 3.38 | 13.1 | 6.82 | － |
| 55 | 6321 | 6328 | 3.38 | 13.1 | 6.82 | － |
| 56 | 6328 | 6333 | 3.38 | 13.1 | 6.82 | － |
| 57 | 6333 | 6339 | 3.38 | 13.1 | 6.82 | － |
| 58 | 6339 | 6345 | 3.38 | 13.1 | 6.82 | － |
| 59 | 6345 | 6346.6 | 3.38 | 13.1 | 6.82 | － |
| 60 | 6346.6 | 6351 | 2.9 | 7.53 | 4.41 | － |
| 61 | 6351 | 6356 | 2.9 | 7.5 | 4.41 | － |
| 62 | 6356 | 6359 | 2.9 | 5.2 | 2.66 | － |
| 63 | 6359 | 6361 | 2.6 | 5.2 | 2.66 | － |
| 64 | 6361 | 6363 | 2.6 | 5.2 | 2.66 | － |
| 65 | 6363 | 6365 | 2.6 | 5.2 | 2.66 | － |
| 66 | 6365 | 6367 | 2.6 | 5.2 | 2.66 | － |
| 67 | 6367 | 6369 | 2.6 | 4.875 | 2.49 | － |
| 68 | 6369 | 6371 | 2.6 | 4.875 | 2.49 | － |
